# The Lens

**Published:** 1873-01-15

**Authors:** 


### "THE LENS;"

A QUARTERLY JOURNAL OF MICROSCOPY AND THE ALLIED NATURAL SCIENCES.

We wish to call the attention of our readers to the fact that a periodical with the above title is published in this city, under the auspices of the State Microscopical Society of Illinois. It is published quarterly, and has just completed its first year. A new volume commenced the present month, and consequently now is a good time to subscribe. It is published in good style, each number containing sixty-four pages, on good type and paper, and is embellished with from one to three engravings or plates in each number.

It is edited by S. A. Briggs, Esq., aided by a publishing committee.

There are but few journals devoted directly to the promotion and diffusion of a knowledge of microscopy published either in this country or in Europe, and hence it is all the more desirable that *The Lens* should be extensively patronized by the cultivators of science generally. At the present time there is no branch of medicine, or of the natural sciences, that does not receive important aid from microscopical investigations. That our readers may judge more accurately of the character and value of *The Lens*, we copy the list of articles in the last number of the year 1872.

The Preparation of Diatomacæ, by Christopher Johnson, M. D.

Reply to Dr. Lionel S. Beale, J. J. Woodward, M. D.

Draw-Tubes *vs.* Deep-Eye-Pieces, by Chas. Stodder.

The Cell. By I. N. Danfurth, M. D.

The Flora of Chicago and Its Vicinity. By H. H. Babcock.

Microscopic Memoranda for the Use of Practitioners of Medicine. By J. J. Woodward, M. D., U. S. A.

The Bailey Collection of Diatomaceæ in the Museum of the Boston Society of Natural History. By Prof. H. L. Smith.

Note on the Frustulia Saxonica as a Test of High-Power Definition. By J. J. Woodward, U. S. A.

Some of the Diatomaceæ of Upper Lake Huron and the Sault. By S. A. Briggs.

Besides these original articles, there are fifteen pages filled with interesting items, gleaned by the editor from various sources.

*The Lens* is published at $3 per annum. Subscriptions and other business matters may be sent to O. S. Wescott, 408 Wabash avenue, Chicago, Illinois.

New York State Medical Society.—The next regular annual meeting of the Medical Society, of the State of New York, will be held at Albany, commencing Feb. 4th, and continuing three days. This is one of the oldest and most efficient State medical societies in the country, and the coming meeting will be one of interest.

